# The NF‐κB/FXR/TonEBP pathway protects renal medullary interstitial cells against hypertonic stress

**DOI:** 10.1111/jcmm.18409

**Published:** 2024-05-21

**Authors:** Chunxiu Du, Shuyuan Hu, Yaqing Li, Hu Xu, Rongfang Qiao, Youfei Guan, Xiaoyan Zhang

**Affiliations:** ^1^ Wuhu Hospital East China Normal University Wuhu Anhui China; ^2^ Health Science Center East China Normal University Shanghai China; ^3^ Division of Nephrology Affiliated Hospital of Nantong University, Medical School of Nantong University Nantong Jiangsu China; ^4^ Advanced Institute for Medical Sciences Dalian Medical University Dalian Liaoning China

**Keywords:** cell viability, FXR, hypertonicity, renal medullary interstitial cells

## Abstract

Farnesoid X receptor (FXR), a ligand‐activated transcription factor, plays an important role in maintaining water homeostasis by up‐regulating aquaporin 2 (AQP2) expression in renal medullary collecting ducts; however, its role in the survival of renal medullary interstitial cells (RMICs) under hypertonic conditions remains unclear. We cultured primary mouse RMICs and found that the FXR was expressed constitutively in RMICs, and that its expression was significantly up‐regulated at both mRNA and protein levels by hypertonic stress. Using luciferase and ChIP assays, we found a potential binding site of nuclear factor kappa‐B (NF‐κB) located in the FXR gene promoter which can be bound and activated by NF‐κB. Moreover, hypertonic stress‐induced cell death in RMICs was significantly attenuated by FXR activation but worsened by FXR inhibition. Furthermore, FXR increased the expression and nuclear translocation of hypertonicity‐induced tonicity‐responsive enhance‐binding protein (TonEBP), the expressions of its downstream target gene sodium myo‐inositol transporter (SMIT), and heat shock protein 70 (HSP70). The present study demonstrates that the NF‐κB/FXR/TonEBP pathway protects RMICs against hypertonic stress.

## 1 BACKGROUND

Farnesoid X receptor (FXR), a member of the nuclear receptor superfamily, is a transcription factor activated by endogenous bile acids. In addition to the liver and small intestine where FXR is highly expressed, the kidney also exhibits highly abundant FXR expression.^1^, ^2^ We previously reported that activation of FXR increases urine concentration by up‐regulating the expression of aquaporin 2 (AQP2)^2^ and that FXR is essential for the survival of renal medullary collecting duct cells (MCDs) under hypertonic stress.^3^ Aside from its important role in water homeostasis, FXR also has protective roles in the development and progression of acute renal injury and chronic kidney diseases.^4^, ^5^

The renal medulla has a special tissue environment in which cells are exposed to extremely high concentrations of sodium chloride, urea, and low oxygen tension.^6^ There are several cell types in renal medulla such as collecting duct epithelial cells, perivascular cells, fibroblasts, and renal medullary interstitial cells (RMICs). RMICs show characteristics of stellate‐like cellular morphology with cytoplasmic projections and abundant cytoplasmic lipid droplets. Previous studies demonstrated that RMICs may play important roles in the maintenance of body fluid homeostasis and normal systemic blood pressure level by synthesizing a vasodepressor lipid called medullipin.^7^, ^8^, ^9^ However, the mechanisms by which RMICs can survive in hypertonic environment remain partially characterized. The tonicity‐responsive enhancer‐binding protein (TonEBP or NFAT5), which belongs to the Rel/NFAT family of transcription factors, is a pleiotropic stress protein that protects cell against hypertonic injury. It is believed that TonEBP plays a critical role in regulatory volume increase (RVI) by controlling the expression of the osmoprotective gene heat‐shock protein 70 (HSP70) and the genes that mediate the intracellular accumulation of small organic osmolytes, among which are aldose reductase (AR), sodium‐chloride‐betaine cotransporter (BGT1), and sodium‐myo‐inositol cotransporter (SMIT).^3^, ^10^ CM Hao et al. have previously reported that water deprivation and hypertonicity can activate NF‐κB and increase cyclooxygenase‐2 (COX‐2) expression to favour RMICs survival in hypertonic conditions.^11^ Moreover, peroxisome proliferator‐activated receptor delta (PPARδ) and glycogen synthase kinase 3β (GSK 3β) have also been reported to be involved in RMIC survival under hypertonic stress.^12^, ^13^

In the present study, we report that FXR is constitutively expressed in RMICs, which are under the same osmotic stress as the collecting duct epithelial cells in the kidney. We found that FXR plays an important role in protecting RMICs from hypertonicity‐induced cell damage via the NF‐κB/FXR/TonEBP pathway.

## 2 METHODS

### 2.1 Chemical reagents and antibodies

Dulbecco's Modified Eagle Medium (DMEM), phosphate‐buffered saline (PBS), fetal bovine serum (FBS), 0.25% (w/v) trypsin–EDTA and penicillin–streptomycin (P/S) were obtained from Gibco (Gaithersburg, MD, USA). Calf serum (CS) was supplied by HyClone (Logan, UT, USA). GW4064 and chenodeoxycholic acid (CDCA) was purchased from Sigma (St Louis, MO, USA). Pierce™ BCA Protein Assay Kit, Lipofectamine 3000 Reagent and SuperSignal West Femto Maximum Sensitivity Substrate were purchased from Thermo Fisher Scientific (Grand Island, NY, USA). The mouse antibody against FXR was purchased from R&D, and the antibodies against pro‐caspase‐3 and cleaved caspase‐3 were obtained from Cell Signalling (Danvers, MA, USA). The antibodies against TonEBP, COX‐2, SMIT, and Caspase‐3 Assay Kit (Fluorometric) were offered by Abcam (Cambridge, MA, USA). Expression vectors of pcDNA, hFXR and NF‐κB (p65) were obtained from Origene (Beijing, China). Triton X‐100 was offered by Bio‐Rad (Hercules, CA, USA).

### 2.2 Animals

Animal experiments were approved by the Ethical Committee of the East China Normal University and conformed to international guidelines for animal usage in research. C57BL/6 mice (male, 8 weeks old) were obtained from Beijing Vital River Laboratory Animal Technology Co. (Beijing, China), housed in a temperature‐controlled room and fed a standard mouse chow. FXR knockout mice were purchased from the Jackson Laboratory.

### 2.3 Primary culture of RMICs

Male mice (8–10 weeks old) were briefly anaesthetised (10 mL/kg, 1% pentobarbital sodium), and Mouse RMICs were cultured as previously reported.^7^ The kidneys were removed and the medulla was dissected and minced under sterile conditions in 6 mL of sterile DMEM supplemented with 16% FBS. The homogenate was injected subcutaneously in the abdominal wall of a mice via a 4‐gauge needle. Three days later, the subcutaneous nodules (Figure S1A) were removed under sterile conditions, cut into 1‐mm fragments, and plated in six‐well plates. The cells were cultured in DMEM medium supplemented with 16% FBS, streptomycin and penicillin (100 U/mL). These cells were characterized and exhibited abundant Oil‐red O‐positive lipid droplets (Figure S1B,C). Cells at passages 2–5 were used. The osmolality of the control medium was 300 mOsm, and the hypertonic medium was generated by adding sodium chloride. When not specifically indicated, the hypertonic medium was prepared by adding sodium chloride to achieve tonicity of 550 mOsm.

### 2.4 Western blot analysis

The protein of RMICs was extracted using an ice‐cold lysis buffer containing phosphatase inhibitor and phenylmethanesulfonyl fluoride. The lysate was homogenized by a sonic oscillator then centrifuged at 12,000 rpm for 10 min at 4°C. Protein concentrations were determined using the Pierce TMBCA Protein Assay Kit. Equal amounts of sample protein (30–60 μg) mixed with 6 × loading buffer were separated by 8%–12% SDS/PAGE gel and transferred to a nitrocellulose membrane. The membrane was incubated with 10% skim milk at room temperature for 1 h for blocking nonspecific binding sites and was then incubated with selected primary antibodies at 4°C overnight. The primary antibodies used in this study were anti‐FXR (1:500), anti‐TonEBP (1:1000), anti‐COX‐2 (1:1000), anti‐β‐actin (1:1000), anti‐pro‐caspase3 (1:1000) and anti‐cleaved caspase‐3 (1:1000). After being washed for 5 min with TBST buffer for five times, the membrane was incubated at room temperature with 1:4000 HRP‐conjugated secondary antibodies (Santa Cruz Biotechnology) for 1 h. The membrane was washed and detected using the SuperLumia ECL Plus HRP Substrate kit (K22030, Abbkine) and the images were collected with Tanon‐5200 (Tanon, Shanghai, China).

### 2.5 Real‐time PCR

Total RNA was extracted from RMICs by using TRIZOL reagent (Biotek), which was then reversed to cDNA using RevertAid™ First Strand cDNA Synthesis Kit (Fermentas, USA) according to the manufacturer's instructions. Real‐time PCR was carried out by using cDNA as a template in the PCR reaction with SYBR Green Mix (Bio‐Rad, Hercules, CA). The reaction conditions included 94°C for 5 min, followed by 35 cycles of 94°C for 30s, 59°C for 30s, 72°C for 30s and a final extension at 72°C for 5 min. β‐actin was used as an internal control. Quantitative values were obtained as the threshold PCR cycle number (Ct). Each sample was measured in duplicate or triplicate in each experiment. The primer pairs used for amplifying interesting mouse genes were listed in Table S1.

### 2.6 Cell viability assay

Cell viability was determined by the MTT assay as previously described.^14^ RMICs were grown to 70%–80% confluence in 24‐well plates and subjected to hypertonic stress for 6 h in the presence and absence of GW4064 (2.5 μM) and CDCA (50 μM). The MTT solution (5 mg/mL) was then added to the medium to reach the final concentration of 0.5 mg/mL. The cells were cultured for another 4 h; at the end of the incubation, the medium was carefully removed and the MTT formazan crystals were dissolved in 500 μL DMSO via incubation in darkness at room temperature for 15 min. Finally, the absorbance was measured at 570 nm by using a microplate reader.

### 2.7 Assay of caspase‐3 activity

Assay of caspase‐3 activity was carried out using a caspase‐3 assay kit (Abcam, ab39383) according to the manufacturer's protocol. RMICs were lysed in pH 7.2 lysis buffer with 10 mM DTT; after incubation for 0.5 h on ice, cell lysate was centrifuged at 12,000 rpm for 30 min at 4°C and the protein concentration in supernatants was measured using the Bradford dye method. Aliquots of 10 mg/100 mL assay volume were treated with 140 mM site‐specific tetrapeptide substrates Ac‐DEVD‐AFC for caspase‐3 in a caspase assay buffer at 37°C with 10 mM DTT for 0.5 h. The release of the fluorogenic group AFC was determined in a microplate reader with excitation at 400 nm and emission at 505 nm. The relative fluorescent units (RFU) were normalized with protein concentrations.^15^

### 2.8 Immunofluorescence staining

RMICs grown to proper confluence were fixed with 4% paraformaldehyde (PFA) at room temperature for 15 min on a rocking platform. After three 5‐min washes with PBS, the cells were permeabilized in 0.1% Triton X‐100 in PBS for 10 min. After being blocked by 5% BSA in PBS for 10 min, the cells were incubated with primary antibodies at 4°C overnight. After washing, the cells were incubated with appropriate dyLight 488 (green) secondary antibodies at 37°C for 30 min. Nuclei were stained with DAPI their images obtained through a confocal microscope.

### 2.9 Luciferase assay

Mouse FXR gene promoter‐driven luciferase reporter was constructed; first, the mouse FXR promoter region was amplified from tail‐derived genomic DNA of C57BL/6 mice. Then, the mouse FXR promoter region containing the fragment −1134 ~ +1 bp was amplified by PCR with the oligonucleotides 5’‐AACTGAGTGACAATGGCAGGT‐3′ (forward primer) and 5′‐ ACTCGGTTCTTCTCTGGGGT‐3′ (reverse primer). The amplified fragment was cloned into the luciferase reporter gene vector PGL3 Basic (Promega, Madison, WI, USA) (Figure S3A), and the resultant construct designated FXR‐luc was sequenced to validate the orientation and sequence (Figure S3B). HEK293T cell line grown to 70–80% confluence was transiently transfected with an FXR‐luciferease reporter plasmid by using Lipofectamine 3000 according to the standard protocols by the manufacturer. The cells were treated with human NF‐κB expression plasmid. β‐galactosidase reporter plasmid was used as a control for transfection efficiency. After 24‐h transfection, the cells were washed twice with cold PBS and lysed with 1 × luciferase lysis buffer (Luciferase Assay Kit, Promega). Luciferase activity was determined by using a luminometer (Turner BioSystems). Luciferase levels of each sample were normalized by β‐galactosidase activity.

### 2.10 Chromatin immunoprecipitation (ChIP) assay

ChIP assay was carried out by a ChIP‐IT® Express Chromatin Immunoprecipitation Kits (Active Motif, Carlsbad, CA, USA). Mouse primary RMICs were seeded in 15 cm plates at a density of 80%–90% confluence. Immunoprecipitation was performed with Anti‐ NF‐κB Antibody (CST) and normal mouse IgG as control at 4°C overnight. Precipitated DNA was analysed by PCR using the primers 5’‐GATCCCCAGTCACCCAACTC‐3′ (sense) and 5’‐TTCCCCTCCCTCCCCTAGA‐3′ (antisense). An amplified product with 219 bp (−1097 bp ~ −878 bp) was then examined by electrophoresis.

### 2.11 Statistical analysis

Statistical analysis was performed using GraphPad Prism 7.0.0 software. Results were presented as means ± standard error of the mean (SEM). Comparisons were performed by Student's *t* test and one‐way ANOVA, with statistical significance set at a *p*‐value of <0.05.

## 3 RESULTS

### 3.1 FXR is constitutively expressed in RMICs

**FIGURE 1:**
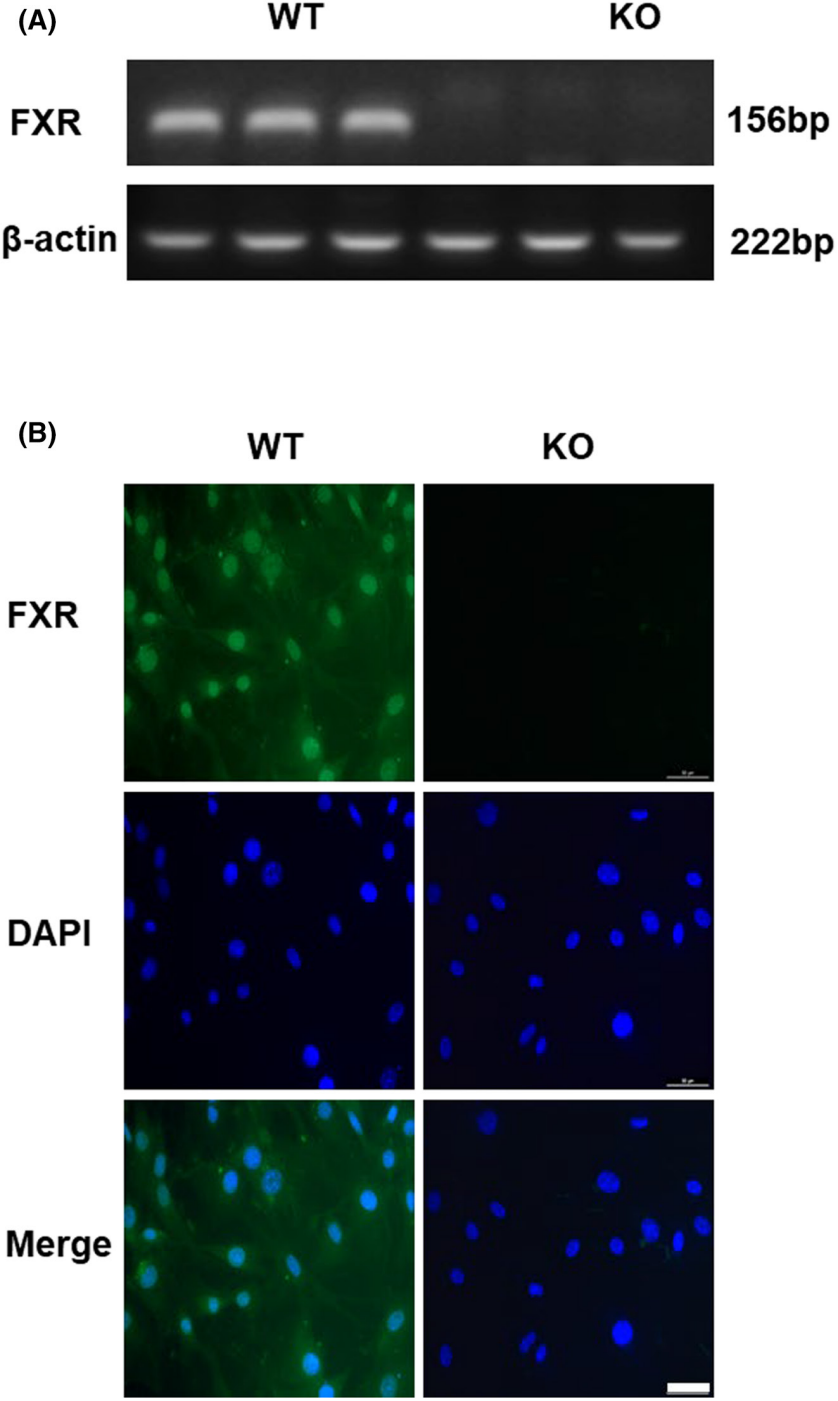
FXR is constitutively expressed in RMICs. Primary RMICs were cultured from wild‐type mice (WT‐RMICs) and FXR gene knockout mice (KO‐RMICs). (A) Real‐time PCR analysis of FXR expression in the WT‐RMICs and KO‐RMICs, *n* = 3. (B) Immunofluorescence staining of FXR (green) in the WT‐RMICs and KO‐RMICs. Note that FXR was mainly expressed in the nucleus (blue). Scale bar is 25 μM.

To identify the expression of FXR in RMICs, we cultured primary RMICs from kidney of wild‐type mice (WT‐RMICs) using FXR gene knockout mice as a negative control (KO‐RMICs). At RNA expression level, we found that while FXR was highly expressed in RMICs, it was not expressed in KO‐RMICs (Figure 1A). We also detected the specific localization of FXR in RMICs by immunofluorescence method and found that FXR was mainly expressed in the nucleus and partly expressed in the cytoplasm of WT‐RMICs, with little signal in KO‐RMICs (Figure 1B). These findings demonstrate that FXR is constitutively expressed in RMICs.

### 3.2 Hypertonicity induces FXR expression in RMICs

**FIGURE 2:**
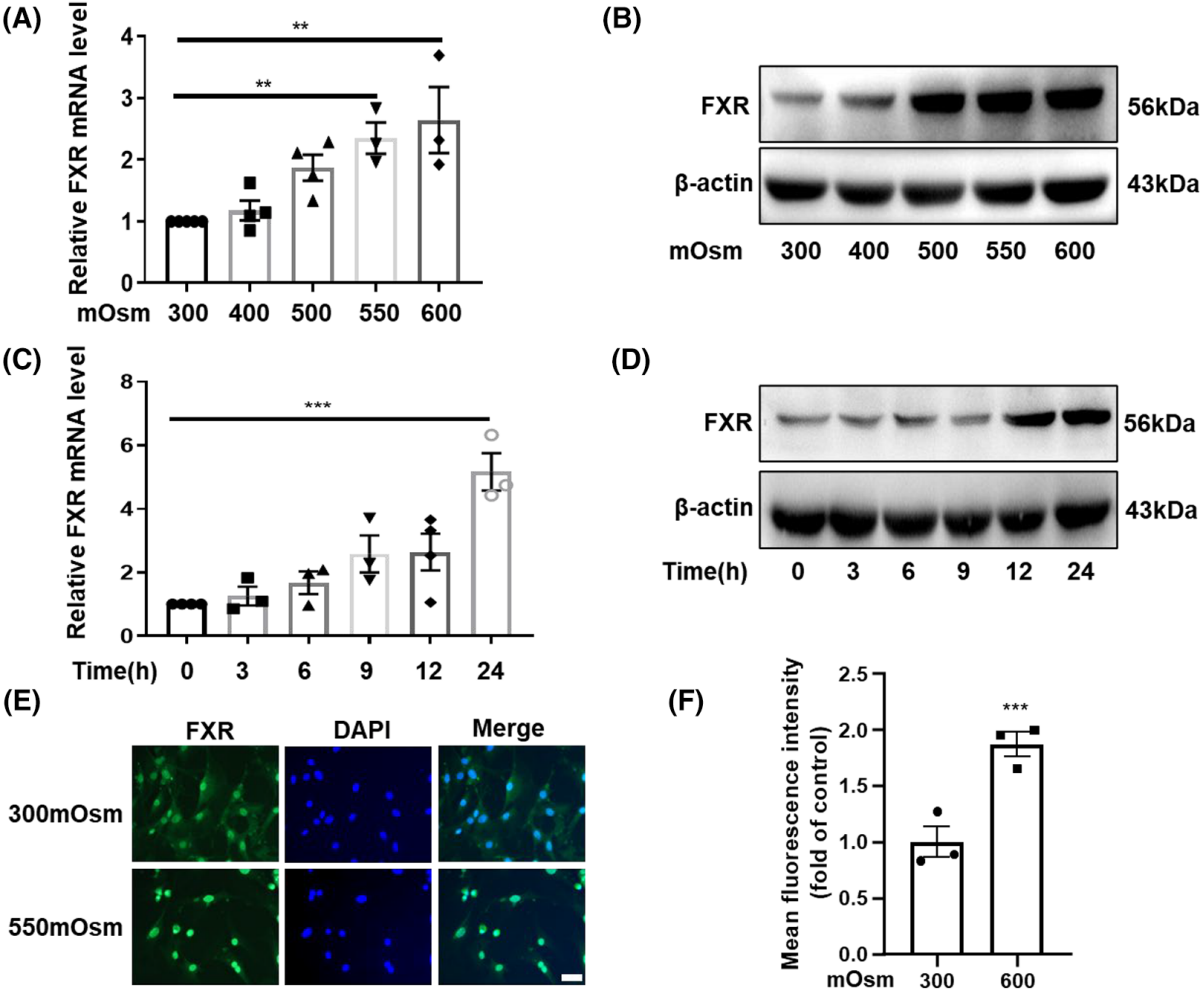
Hypertonicity induces FXR expression in RMICs. (A) Expression levels of FXR in the primary cultured RMICs. Cells were treated with hypertonic solution in a dose‐dependent manner for 12 h. **p < 0.01, *n* = 3–4. (B) Representative western blot analysis of FXR expression in RMICs exposed to various hypertonicity. (C) Expression levels of FXR in the RMICs. Cells were treated with hypertonic solution (550 mOsm) in a time‐dependent manner. ***p < 0.001, *n* = 3–4. (D) Representative western blot analysis of FXR expression in RMICs treated with 550 mOsm at various time points. (E) Immunofluorescence was performed to detect FXR (green) expression and nuclear translocation in RMICs. The nucleus was visualized by staining with DAPI (blue). Scale bar is 25 μm. (F) Quantitative analyses of FXR fluorescence in Figure 2E. ***p < 0.001, *n* = 4.

RMICs live in a harsh environment with high osmolality and low oxygen tension. To further explore the role of FXR in the survival of RMICs, we determined the expression of FXR in RMICs under hypertonicity. We treated mouse primary RMICs with high osmotic pressure in a dose‐dependent manner then measured mRNA and protein levels of FXR. The results showed that the mRNA levels of FXR (Figure 2A) were upregulated as the osmotic pressure increased. Similarly, the protein levels of FXR were also markedly elevated by hypertonicity (Figure 2B). Meanwhile, we also treated mouse primary RMICs with 550 mOsm in a time‐dependent manner and found that both mRNA and protein levels of FXR were induced by hypertonicity (Figure 2C,D). Furthermore, hypertonicity treatment resulted in a robust nuclear translocation of FXR after exposure to 550 mOsm for 12 h (Figure 2E,F), which indicates that FXR might be involved in anti‐hyperosmotic stress in RMICs. As TonEBP, COX‐2 and NF‐κB have been previously reported to play a critical role in maintaining the survival of RMICs under hypertonic stress,^16^, ^17^ we therefore determined their expressions in our cell culture system. In line with previous findings, our results showed that hypertonicity also markedly induced gene expressions of TonEBP, COX‐2 and NF‐κB in RMICs in a dose‐dependent manner (Figure S2A–D). Collectively, our results demonstrate that hypertonicity induces FXR and important osmoprotective gene expression in RMICs, suggesting that FXR may also be a hypertonicity responsive gene that might be involved in cell viability regulation in RMICs under hypertonic stress.

### 3.3 Transcription of FXR is promoted by NF‐κB

**FIGURE 3:**
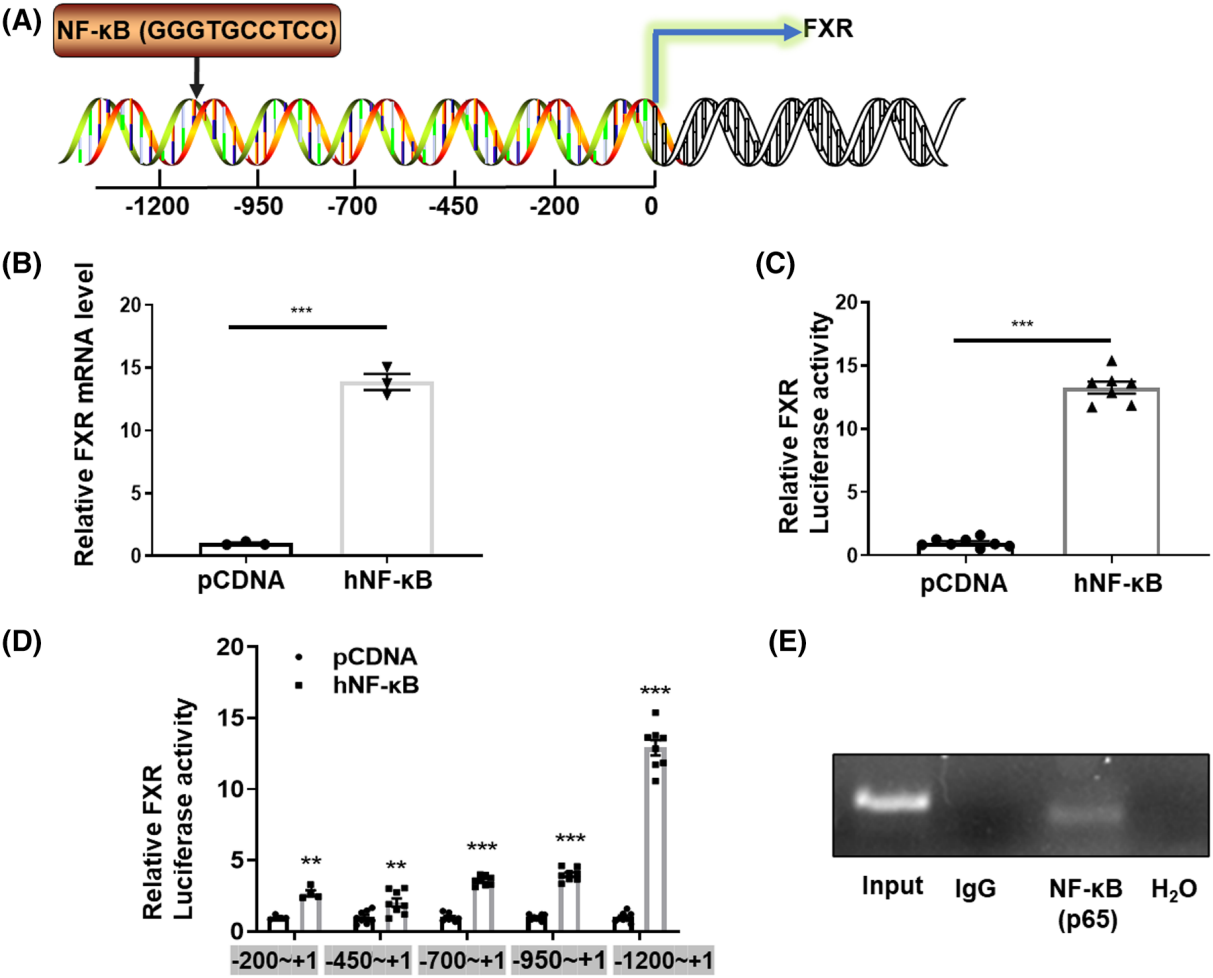
FXR transcription is promoted by NF‐κB. (A) DNA sequence analysis revealed a potential NF‐κB binding site within the promoter region of mouse FXR gene located between −1134 bp and − 884 bp upstream from the transcription start site. (B) Real‐time PCR analysis demonstrates the effect of human NF‐κB overexpression on mRNA expression of FXR in RMICs. *** *p* < 0.001, *n* = 3. (C) Luciferase activity was measured to assess the transcription activity of FXR gene. HEK293T cells were transfected with the pcDNA plasmid, FXR‐luc, human NF‐κB expression vector for 24 h. ***p < 0.001, *n* = 7–8. (D) Luciferase assay revealed the effect of human NF‐κB overexpression on FXR gene promoter activity using the luciferase reporters driven by truncated FXR gene promoter fragments including −1200 ~ +1, −950 ~ +1, −700 ~ +1, −450 ~ +1, −200 ~ +1. ** *p* < 0.01, *** *p* < 0.001, *n* = 4–8. Data are presented as mean ± SEM. (E) ChIP assay revealed that NF‐κB (p65) can bind to the predicted NF‐κB binding site in mouse FXR gene promoter in primary RMICs. Input, positive control; IgG, IgG precipitated DNA as negative control; NF‐κB anti‐NF‐κB antibody precipitated DNA; H_2_O, blank control.

To characterize the underlying mechanism responsible for hyperosmotic‐induced FXR expression, we tested the role of NF‐κB, an important transcription factor upregulated by hypertonicity and critical for the survival of RMICs.^17^ We speculated that NF‐κB may bind to the NF‐κB binding elements existing in the FXR gene promoter as a transcription factor. We used the Transcription Element Search System (TESS) to analyse a 2 kb sequence upstream of the mouse FXR gene transcription start site. We found a potential NF‐κB binding site (5’‐GGGTGCCTCC‐3′) located between −1134 bp and − 884 bp upstream from the transcription start site of the FXR gene then constructed a FXR gene promoter‐driven luciferase reporter gene (FXR‐Luc) using the pGL3‐Basic vector (Figure 3A). We found that human NF‐κB overexpression significantly increased FXR mRNA levels in primary cultured RMICs (Figure 3B). Similarly, FXR‐Luc activity was also significantly activated by human NF‐κB overexpression in HEK293T cell lines (Figure 3C). To further verify the specific NF‐κB binding site in the FXR promoter region, we truncated the FXR promoter sequence and constructed four luciferase reporters driven by various promoter regions. We found that the up‐regulation effect of NF‐κB on the FXR‐Luc was almost abolished after deleting the sequence of nt1200−nt950 (Figure 3D), which contains the potential NF‐κB binding element (5’‐GGGAATTTCC‐3′) (Figure 3A). The ChIP assay further revealed a single band with the predicted size, indicating that NF‐κB can directly bind to the NF‐κB binding element located in the FXR gene promoter (Figure 3E). These results demonstrate that NF‐κB may play an important role in hypertonicity‐induced FXR expression in RIMCs.

### 3.4 FXR protected rmics from hypertonicity‐induced cell apoptosis

**FIGURE 4:**
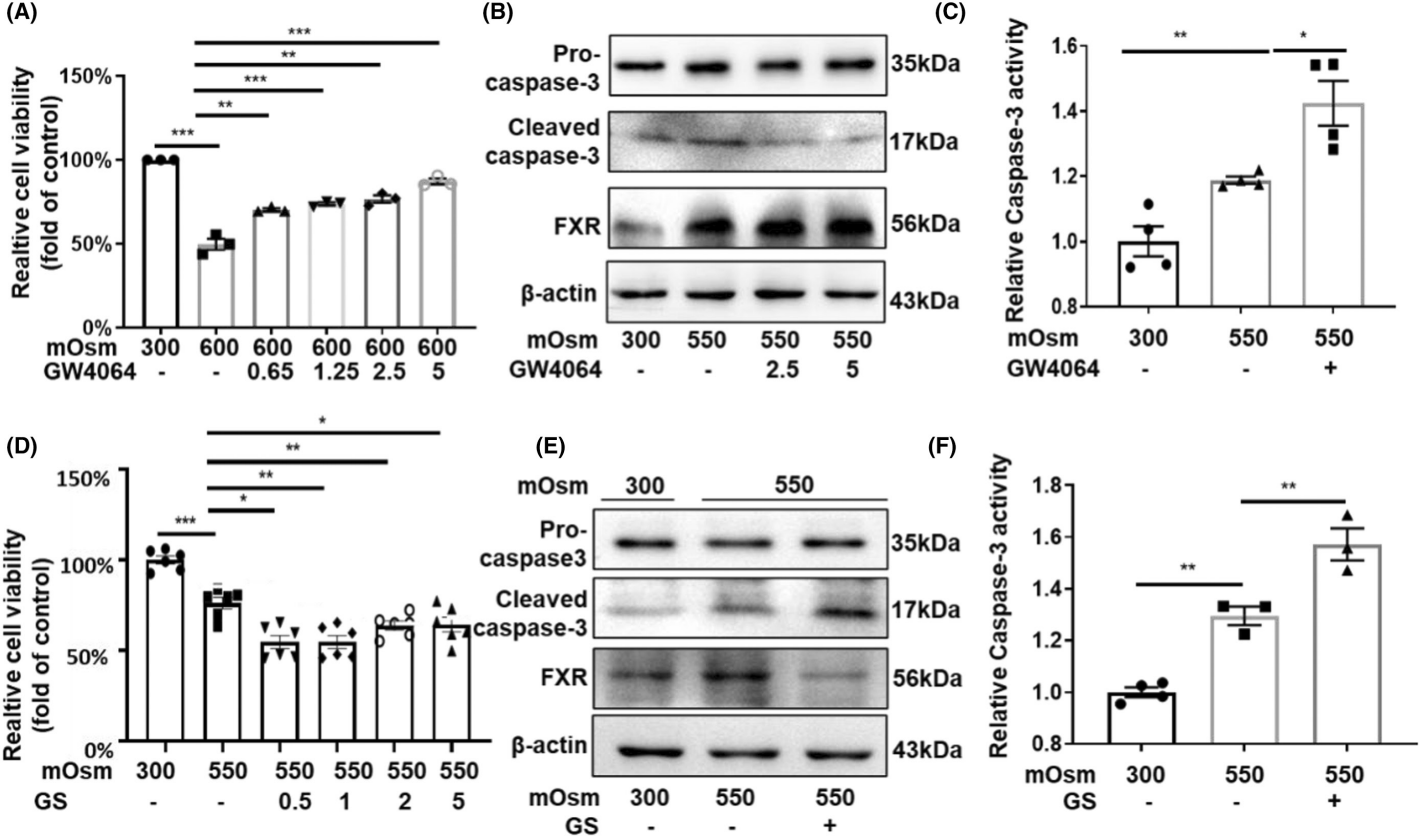
FXR protects RMICs from hypertonicity‐induced cell apoptosis. (A) MTT cell viability analysis demonstrated the protective effect of FXR activation on hypertonicity‐induced cell death of RMICs. Cells were pretreated with the FXR agonist GW4064 in a dose‐dependent manner for 12 h and then treated with hypertonic solution (600 mOsm) for another 12 h. **p<0.01, *** *p* < 0.001, *n* = 3. (B) Western blot analysis of protein expression of caspase‐3 and FXR in RMICs treated with and without GW4064 (2.5, 5 μM) under isosmotic and hyperosmotic pressure (550 mOsm). (C). Caspase‐3 activity analysis in the RMICs treated with and without GW4064 (5 μM) under isosmotic and hyperosmotic pressure (550 mOsm). *p<0.05, ** *p* < 0.01, *n* = 4. (D) MTT cell viability analysis demonstrated that FXR inhibition increased hypertonicity‐induced cell death of RMICs. Cells were pretreated with the FXR inhibitor guggulsterone (GS) in a dose‐dependent manner for 12 h and then treated with hypertonic solution (550 mOsm) for another 12 h. *p<0.05, **p < 0.01, ***p<0.001, *n* = 6. (E, F) GS can enhance the protein expression and activity of caspase‐3, an indicator of apoptosis, under hypertonic conditions. **p < 0.01, *n* = 3–4.

To verify whether FXR promotes the survival of RMICs in hypertonic environment, we treated RMICs with GW4064, a specific agonist of FXR, in a dose‐dependent manner (0, 0.65, 1.25, 2.5, 5 μM) for 24 h then cultured the cells with hypertonic medium (600 mOsm) for an additional 12 h. The cell viability was measured by the MTT assay, and we found that hypertonicity decreased cell viability while GW4064 increased the survival of RMICs under hypertonicity (Figure 4A). Cleaved caspase‐3 is an active form of pro‐caspase‐3, which is the best‐known marker of apoptosis.^18^ The western blot results showed that FXR activation markedly attenuated the protein levels of cleaved caspase‐3 in RMICs exposed to hyperosmotic stress (Figure 4B). Similarly, treatment with the FXR agonist GW4064 significantly inhibited the elevation of caspase‐3 activity induced by hyperosmolality in RMICs (Figure 4C). In contrast, the FXR inhibitor guggulsterone (GS) significantly aggravated cell damage in a dose‐dependent manner under hypertonic conditions (Figure 4D). Furthermore, GS enhanced the protein expression of the cleaved caspase‐3 (Figure 4E) and activity of caspase‐3 under hypertonic conditions (Figure 4F). These findings indicate that FXR activation can promote the survival of RMICs in hyperosmotic condition, while inhibition of FXR can aggravate apoptosis of RMICs.

### 3.5 FXR activation enhanced the expression and nuclear translocation of TonEBP in cultured RMICs

**FIGURE 5:**
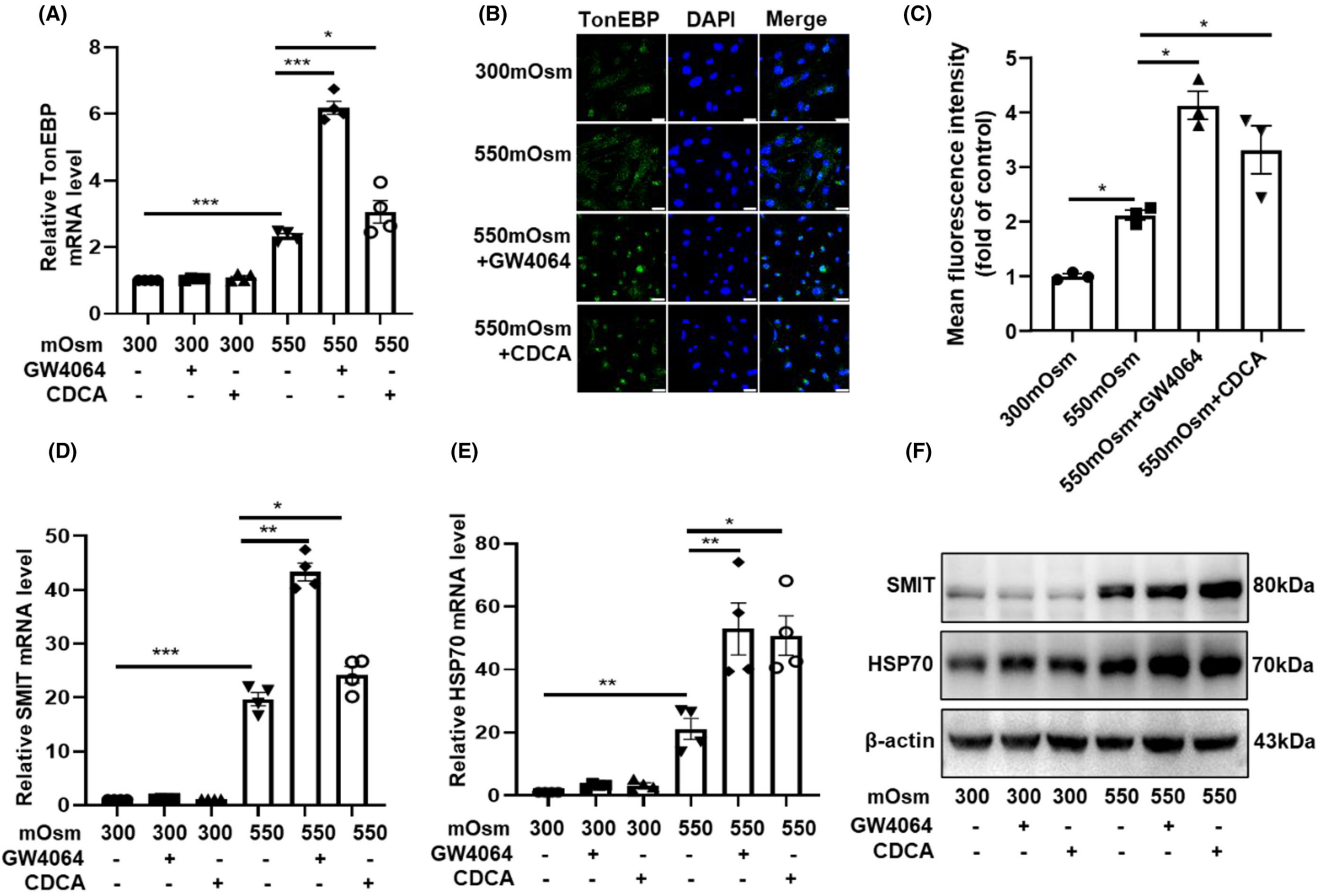
FXR activation enhances TonEBP expression and nuclear translocation in cultured RMICs. (A) Real‐time PCR analysis demonstrating that the FXR activation increased the mRNA expression of TonEBP. RMICs were treated with DMSO, GW4064 or CDCA for 24 h and then subjected to hypertonic solution (550 mOsm) for 12 h. *p < 0.05, ***p < 0.001, *n* = 4. (B) Immunofluorescence study showed that FXR activation enhanced nuclear localization of TonEBP (green) in RMICs. Cells were pretreated with the FXR agonist CDCA (75 μM) or GW4064 (5 μM) for 12 h and then treated with hypertonic solution (550 mOsm) for 12 h. The nucleus was visualized by staining with DAPI (blue). Scale bar is 25 μm. (C) Quantitative analyses of TonEBP fluorescence in Figure 5B. *p < 0.05, *n* = 3. (D, E) Quantitative PCR showing that FXR activation by GW4064 and CDCA increased the mRNA expression of the TonEBP downstream gene SMIT and HSP70 in RMICs under hypertonic condition. *p < 0.05, **p < 0.01, *** *p* < 0.001, *n* = 4. (F) Western blot assay showed the protein levels of SMIT and HSP70 in RMICs treated with FXR agonists under hypertonicity.

Previously, we have reported that FXR protects the epithelial cells of renal collecting ducts against hyperosmotic injury by inducing TonEBP expression and translocation.^3^ TonEBP is a classical osmotic regulator which increases the transcription of target genes whose protein products are involved in the accumulation of organic osmolytes, including glycine betaine, myo‐inositol, glycerophosphocholine and sorbitol, leading to osmoprotection. Accordingly, we pre‐treated the primary RMICs with GW4064 and CDCA (an endogenous agonist of FXR) for 12 h then cultured the cells with hypertonic medium (550 mOsm) for another 12 h. We found that the mRNA levels of TonEBP were significantly upregulated by hypertonicity (Figure S2; Figure 5A). Activation of FXR by GW4064 and CDCA further enhanced hypertonicity‐induced TonEBP mRNA expression (Figure 5A). Similarly, FXR activation also markedly increased the nuclear translocation of TonEBP (Figure 5B,C), suggesting an increase in TonEBP activity. As expected, the hypertonicity‐induced mRNA and protein expression of the downstream target genes of TonEBP (SMIT and HSP70) were further increased after FXR activation (Figure 5D–F). Together, these findings suggest that TonEBP may mediate the cytoprotective effect of FXR in RMICs against hypertonic injury.

## 4 CONCLUSION

**FIGURE 6:**
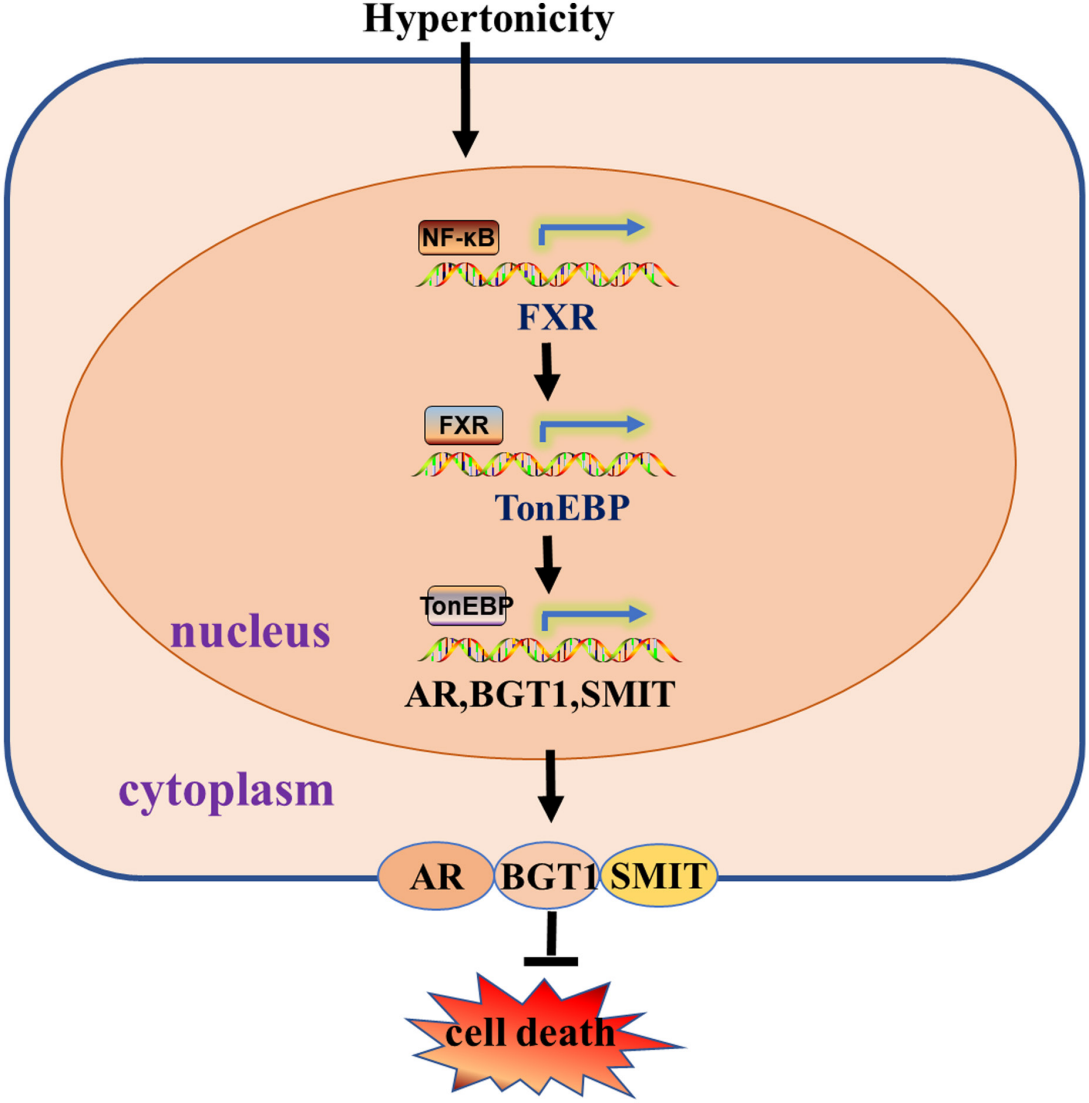
Schematic showing the underlying mechanism by which the NF‐κB /FXR/ TonEBP pathway protects renal medullary interstitial cells against hypertonic stress. Under hypertonic conditions, NF‐κB binds to the promoter region of FXR to promote the increase of FXR transcription, then FXR binds to the promoter region of TonEBP to promote the increase of TonEBP transcription. Finally, TonEBP inhibits cell death under hypertonic conditions by increasing the transcription of AR, BGT1, SMIT.

This study demonstrates that hypertonic stress increases FXR transcription at least in part through NF‐κB. FXR activation is essential for the survival of RMICs in this hostile condition. We have confirmed that FXR is constitutively expressed in primary cultured RMICs, and that its expression and activation are significantly increased by hypertonicity treatment. In primary cultured RMICs, activation of FXR markedly promotes cell viability. In contrast, inhibition of FXR activity aggravates cell apoptosis of RMICs under hypertonicity. We have also provided evidence that expression and activity of TonEBP are significantly increased in response to FXR activation under hypertonic stimulation. Sequence analysis of the FXR gene promoter identifies a classical binding site of NF‐κB, which can be bound by NF‐κB leading to a significant increase in FXR gene transcription. Collectively, our findings demonstrate that the NF‐κB /FXR/ TonEBP pathway protects RMICs against hypertonic stress‐induced cell death (Figure 6).

FXR belongs to the superfamily of nuclear receptor transcription factors and can be activated by many naturally occurring endogenous bile acids.^3^, ^19^ Like many other nuclear receptors, FXR regulates the transcription of target genes by binding to a FXR response element (FXRE) as a monomer or heterodimer with the retinoid X receptor, another nuclear receptor.^20^, ^21^ FXR is highly expressed in the liver and small intestine, where it is essential in the regulation of bile acid metabolism and enterohepatic circulation.^22^ The kidney has constitutively high expression of FXR, with a ubiquitous expression pattern along renal tubules.^23^ Increasing evidence demonstrates that activation of FXR improves diabetic tubular function and tubular toxicity.^23^, ^24^ Our previous studies demonstrated that disruption of FXR gene causes a significant defect in urine concentrating ability, resulting in a polyuria phenotype in mice. FXR activation increases AQP2 expression and FXR gene deficiency significantly potentiate MCDs death in mice with water restriction.^2^, ^3^ However, the function of FXR in RMICs remains largely uncharacterized. In the present study, we found that FXR is protective in RMICs exposed to hypertonic stress, possibly by increasing the expression and nuclear translocation of TonEBP.

Currently, multiple osmo‐protective mechanisms have been reported to participate in maintaining the survival of RMICs under hypertonic stress. Moeckel et al. demonstrated that COX‐2 activity promotes organic osmolyte accumulation and adaptation of RMICs to hypertonic stress.^8^ Hao CM et al. discovered that dehydration activates an NF‐κB‐driven, COX‐2‐dependent survival mechanism in RMICs. In addition, PPARδ activation is also involved in the physiological response of RMICs to environmental osmotic stress.^11^, ^25^ Our results suggest that FXR may represent a novel hypertonicity responsive gene like TonEBP. Increasing evidence suggests that NF‐κB as a master regulator of inflammation is critical in maintaining renal medullary cell viability under water deprivation and hypertonicity conditions.^11^, ^26^ The present study showed that NF‐κB can directly bind to the promoter region of the FXR gene, thereby activating an NF‐κB‐driven, FXR‐dependent survival mechanism in RMICs. To the best of our knowledge, this is the first time that NF‐κB has been found to be a transcriptional regulator of the FXR gene in RMICs. Previously, we have reported that the serine/threonine kinase 5′‐AMP protein kinase (AMPK) activation results in reduced cell viability, possibly by blocking the NF‐κB‐COX‐2‐Prostanoid survival pathway.^11^, ^17^ Considering that FXR is a downstream target gene of NF‐κB, the question as to whether it can affect COX‐2 expression and prostaglanoid production remains unclarified. Addressing these important issues may significantly advance our knowledge in understanding the mechanism by which FXR is regulated and the importance of FXR in renal physiology.

In summary, the present study demonstrates that FXR is essential for the survival of RMICs under hypertonic stress. Hypertonicity increases FXR expression and its transcriptional activity in RMICs. NF‐κB plays a critical role in inducing FXR expression under hypertonic condition, which protects against apoptotic cell death of RMICs. Furthermore, FXR activation exhibits osmoprotective effect by promoting TonEBP expression and nuclear translocation in RMICs. Our findings provide a novel mechanism by which the NF‐κB/FXR/TonEBP axis contributes to the survival and function of RMICs in dehydrated state.

## AUTHOR CONTRIBUTIONS

**Chunxiu Du:** Formal analysis (equal); investigation (equal); methodology (equal); project administration (equal); software (equal); validation (equal); writing – original draft (equal). **Shuyuan Hu:** Validation (equal). **Yaqing Li:** Validation (equal). **Hu Xu:** Supervision (equal). **Rongfang Qiao:** Software (equal). **Youfei Guan:** Funding acquisition (equal); supervision (equal); writing – review and editing (equal). **Xiaoyan Zhang:** Funding acquisition (equal); methodology (equal); project administration (equal); supervision (equal); writing – review and editing (equal).

## FUNDING INFORMATION

National Natural Science Foundation of China 82270703 to Xiaoyan Zhang. National Natural Science Foundation of China 81970606 to Xiaoyan Zhang. National Natural Science Foundation of China 81970595 to Youfei Guan. East China Normal University 2022JKXYD03001 to Xiaoyan Zhang.

## CONFLICT OF INTEREST STATEMENT

The authors declare no conflicts of interest.

## CONSENT

The author confirms that the work described has not been published before and its publication has been approved by all co‐authors.

## Supporting information


Data S1.

## Data Availability

The data that support the findings of this study are available from the corresponding author upon reasonable request.
