# Supplementary material for: The NF‐κB/FXR/TonEBP pathway protects renal medullary interstitial cells against hypertonic stress
Source: J Cell Mol Med. 2024 May 21;28(10):e18409. doi: 10.1111/jcmm.18409 (PMC11106643; doi:10.1111/jcmm.18409)
Supplement: Supplementary file 1 — Data S1. [file JCMM-28-e18409-s001.docx]

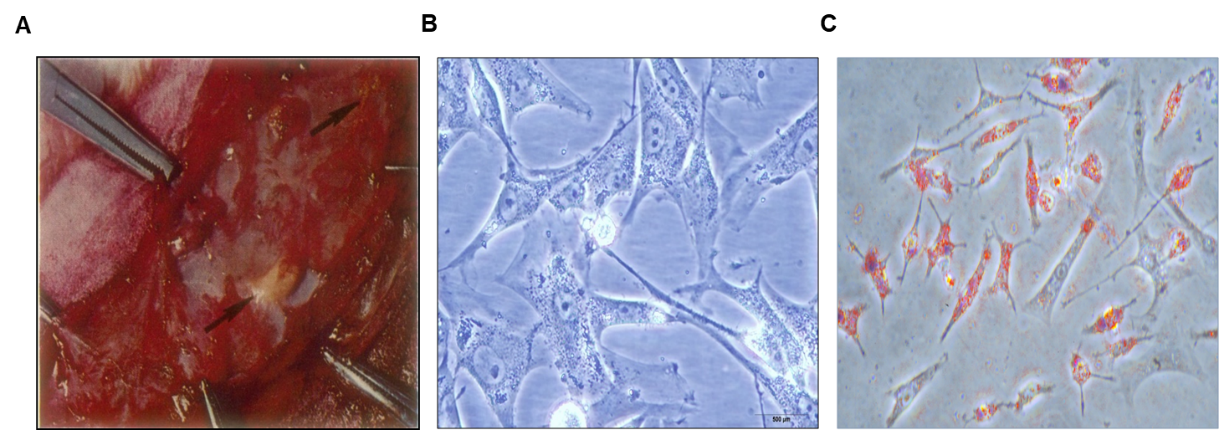


**Supplemental Figure 1.** Primary culture and validation of mouse RMICs. (A) Photograph showed the subcutaneous nodules of medulla nephrica. (B) Under the light microscope, RMICs were characterized as long fusiform with irregular starburst and exhibited many lipid droplets. Magnification 400X. (C) The RMICs were verified by positive Oil red O staining. Magnification 100X.

**
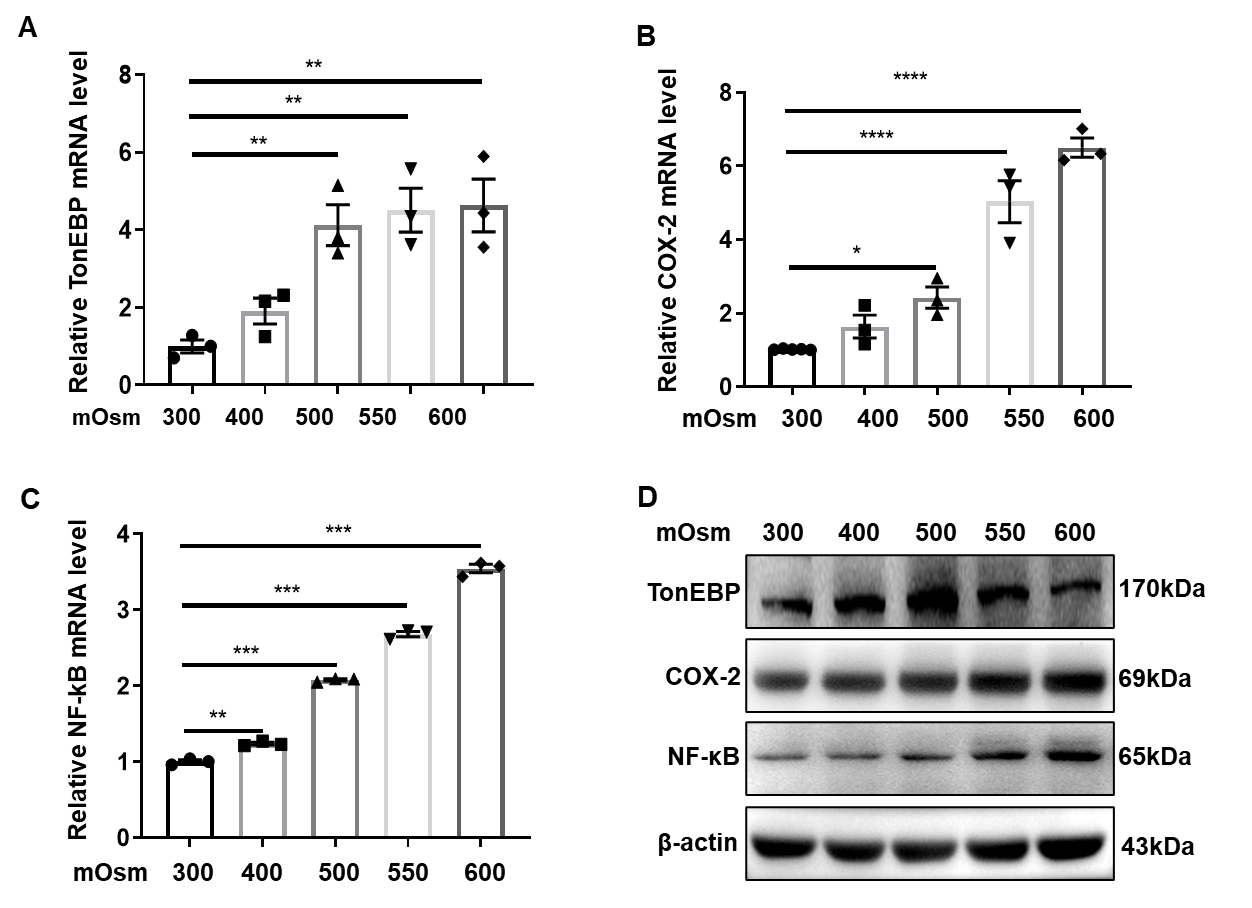
**

**Supplemental Figure 2.** Hypertonicity induces osmoprotective genes expression in RMICs. (A-C) Expression levels of TonEBP, COX-2 and NF-κB in the primary cultured RMICs. Cells were treated with hypertonic solution in a dose-dependent manner for 12 h. * *P* < 0.05, ** *P* < 0.01, *** *P* < 0.001, **** *P* < 0.0001. n =3-4. (D) Representative Western blot analysis of TonEBP, COX-2 and NF-κB expression in RMICs cultured in the solutions with various hypertonicity. Supplementary Figure 2D and Figure 2B used the same samples, thus the same β-actin blot was used as loading control.


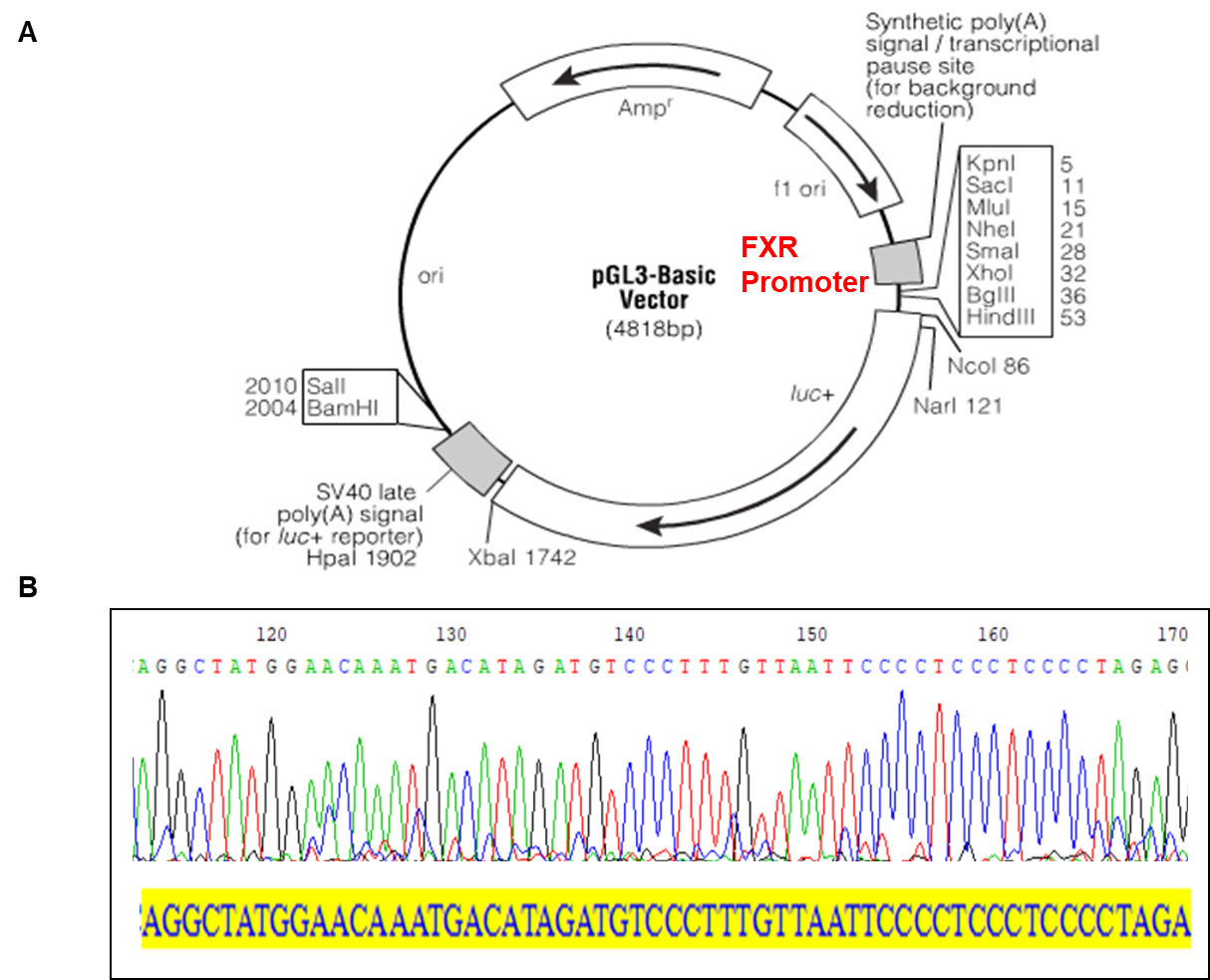


**Supplemental Figure 3.** Construction of FXR gene promoter-driven luciferase Reporter (FXR-Luc). (A) Schematic of the FXR-Luc reporter. Restriction enzymes were used to subclone the FXR promoter DNA sequence using the pGL3-basic vector to construct FXR-Luc. (B) The FXR-Luc was validated by DNA sequencing. The sequence of the FXR-Luc (upper panel) is identical to the mouse FXR promoter sequence (lower panel).

| **Supplemental Table 1. Primer pairs used for amplifying mouse genes.** | |
| --- | --- |
| Name | Sequences |
| β-actin forward | 5’ AGCCATGTACGTAGCCATCC 3’ |
| β-actin reverse | 5’ GCTGTGGTGGTGAAGCTGTA 3’ |
| FXR forward | 5’ TGGGTACCAGGGAGAGACTG 3’ |
| FXR reverse | 5’ CGGAAGAAACCTTTGCAGCC 3’ |
| TonEBP forward | 5’ ACCTCTTCCAGCCCTACCAT 3’ |
| TonEBP reverse | 5’ CTTCGGGGTTGATGGATGCT 3’ |
| NF-κB forward | 5’ AAACCTCGCGTTGATCTCGA 3’ |
| NF-κB reverse | 5’ GACTTGCGGTTCTTCTCGCT 3’ |
| AR forward | 5’ GACACTTGGACGGCTATGGA 3’ |
| AR reverse | 5’ ATGCCTTTGCTGTGGCAGTA 3’ |
| HSP70 forward | 5’ CCGACAAGGAGGAGTTCGTG 3’ |
| HSP70 reverse | 5’ ACAGTAATCGGTGCCCAAGC 3’ |
| COX-2 forward | 5’ CACTCTATCACTGGCACCCC 3’ |
| COX-2 reverse | 5’ TTGGCACATTTCTTCCCCCA 3’ |
